# Study Design and Baseline Profiles of Participants in the Tianjin Birth Cohort (TJBC) in China

**DOI:** 10.2188/jea.JE20200238

**Published:** 2022-01-05

**Authors:** Shuo Wang, Guohong Zhang, Jing Wang, Zhiqiang Ye, Huikun Liu, Lingyao Guan, Yijuan Qiao, Jiayu Chen, Tao Zhang, Qian Zhao, Yu Zhang, Bo Wang, Ya Gao, Puyi Qian, Lingyan Feng, Fang Chen, Gongshu Liu

**Affiliations:** 1 Tianjin Women’s and Children’s Health Center, Tianjin, China; 2 BGI-Shenzhen, Shenzhen, China; 3 China National GeneBank, Shenzhen, China

**Keywords:** design, birth cohort, child, early-onset chronic disease, biobank

## Abstract

**Background:**

To investigate the causal link between early-life exposures and long-term health consequences, we established the Tianjin Birth Cohort (TJBC), a large-scale prospective cohort in northern China.

**Methods:**

TJBC aims to enroll 10,000 families with follow-ups from pregnancy until children’s six year-old. Pregnant women and their spouses were recruited through a three-tier antenatal healthcare system at early pregnancy, with follow-ups at mid-pregnancy, late pregnancy, delivery, 42 days after delivery, 6 months after delivery, and each year until 6 years old. Antenatal/neonatal examination, biological samples and questionnaires were collected.

**Results:**

From August 2017 to January 2019, a total of 3,924 pregnant women have already been enrolled, and 1,697 women have given birth. We observed the prevalence of gestational diabetes mellitus as 18.1%, anemia as 20.4%, and thyroid hypofunction as 2.0%. In singleton live births, 5.6% were preterm birth (PTB), 3.7% were low birth weight, and 7.3% were macrosomia. Based on current data, we also identified maternal/paternal factors which increased the risk of PTB, including paternal age (OR 1.07; 95% CI, 1.01–1.14 for each year increase), vaginal bleeding during pregnancy (OR 2.82; 95% CI, 1.54–5.17) and maternal early-pregnancy BMI (OR 1.08; 95% CI, 1.01–1.15 for each kg/m^2^ increase).

**Conclusion:**

TJBC has the strength of collecting comprehensive maternal, paternal, and childhood information. With a diverse range of biological samples, we are also engaging with emerging new technologies for multi-omics research. The study would provide new insight into the causal link between macro/micro-environmental exposures of early life and short/long-term health consequences.

## INTRODUCTION

In the past decades, China experienced great improvement in health conditions as a result of enormous social and economic development. The mortality rate under 5 years old and the maternal mortality rate reduced with an average annual rate of 8.2%^1^ and 8.9% from 1996 to 2015.^2^ However, rapid economic growth was also accompanied with new health challenges, including exposing to changing factors such as lifestyle patterns (especially dietary behaviors),^3^ pollution, late marriage and childbearing, smoking and drinking, and birth policies. Correspondingly, those exposures might lead to changes in omics biomarkers, such as metabolic molecules, gut microbes, cell-free RNA, and cell-free DNA. Eventually, there has been a great transition of disease spectrums, including the surge of chronic non-communicable diseases,^4^ infertility,^5^ and increased growth and development problems in children.^6^ According to the theory of the Developmental Origins of Health and Disease (DOHaD), maternal/paternal exposures during pregnancy can have important roles in the change of the short-term and long-term health consequences of the offspring.^7^^,^^8^ It can be foreseen that the drastic social and economic development in China, and the resulting changes in people’s behaviors and psychology, will have a profound impact on the health of mother and child in China in the next several decades. To better understand the social and economic impacts on maternal and child health, and identify key exposures affecting children long-term development, we intend to establish a large prospective birth cohort in Tianjin, a northern metropolitan city of China, that continuously collects longitudinal epidemiological data, biological samples and phenotype information from early pregnancy to children at 6 years old.

We established the Tianjin Birth Cohort (TJBC) based on the following reasons: (1) Tianjin has been exposed to complex environmental and social factors that may have significant impacts on maternal and child health. For instance, Tianjin is one of the cities most affected by air pollution as a result of rapid industrialization and urbanization. It is also a typical city in China facing the problems of population aging and advanced maternal age; (2) Tianjin has developed a three-tier antenatal healthcare system, which is effective in organizing population-based cohort projects; (3) Although several birth cohorts have been set up in Guangzhou and several other cities^9^^–^^11^^,^^20^ in China, there is a lack of a large cohort in the northern part of China. Tianjin is 120 kilometers away from Beijing, with a relatively developed economy^12^ and large population, which provides a good foundation for large-scale population studies in northern China; (4) Tianjin has regional health problems, such as high prevalence of gestational diabetes mellitus (GDM; 9.3% in 2012)^13^ and childhood obesity (16.3% in 2014) comparing with other Chinese cities.^14^ There is an imminent demand to determine public health policies to cope with future health challenges. Therefore, Tianjin is a unique and important place to investigate the health impacts of early-life exposures on fetal development, childhood growth, and long-term disease onset.

Latest research showed that multi-omics technology was of great significance in disease prediction and precision medicine. A longitudinal big data study conducted at Stanford University School of Medicine showed that multi-omics research greatly improved the predictive performance of diabetes, cardiovascular disease, tumors, and other diseases compared with clinical data alone.^22^ Researchers at Baylor College of Medicine also found that multi-omics technology may have the potential to extend healthy life among active adults through improved prevention and early detection of age-related chronic diseases.^23^ At present, most studies on pregnant complications and childhood diseases were focused on single-level omics,^24^^,^^25^ but for those complex diseases each technology performed only limited analyses for the etiology of the disease. Therefore, in the current study, we hypothesized that longitudinal multi-omics profiling combined with early exposures may improve disease risk prediction of pregnant complications and childhood diseases such as childhood obesity, hypertension, autism, and allergy.

The research interests of the TJBC will focus on identifying the biomarkers of severe pregnant complications (such as GDM, pre-eclampsia, pre-term birth, and stillbirth) and birth defects using multi-omics technology (eg, genome, transcriptome, metabolome, and microbiome), following the association study between disease biomarkers and clinical data (including the height, weight, clinical test results, ultrasound measurements, pregnancy complications, and medication). More importantly, we will use the TJBC to explore the roles of early exposures during pregnancy in determining children developmental disorders (eg, mental retardation, autism, and allergy) and children’s early-onset chronic diseases (eg, childhood obesity, hypertension, diabetes, and dyslipidemia).

## METHODS

### Participants and follow-up

**Figure 1.**
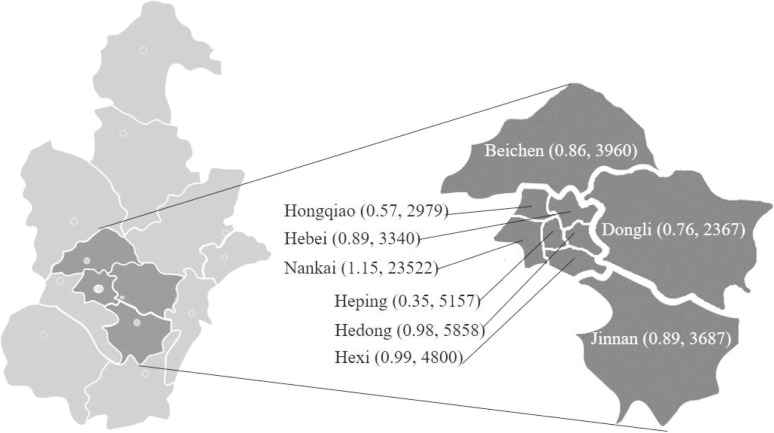
Population and annual births of infants of the study setting (in million people and births/year)

The TJBC was a population-based birth cohort, aiming to recruit 10,000 families with follow-ups from early pregnancy until children are 6 years old. The study recruited women and their spouses at early pregnancy from six central urban districts (Heping, Nankai, Hexi, Hedong, Hebei, and Hongqiao) and three suburban districts (Beichen, Dongli, and Jinnan) of Tianjin, covering annual birth of 55,670 infants (Figure 1). Pregnant women and their spouses were recruited through an integrated three-tier antenatal healthcare system, consisting of (1) Community-level primary healthcare centers; (2) District-level Women and Children’s Health Centres (WCHC) and other secondary obstetric hospitals; and (3) A city-level Tianjin WCHC (TWCHC) and other tertiary hospitals, which has been described in detail previously.^21^ First, all pregnant women who set up pregnancy records in the nine districts were informed about the cohort verbally or through propaganda posters; then, eligible individuals (together with their spouses) who were willing to participate would be referred to district-level WCHCs for recruitment and baseline investigation. And TWCHC administrates and provides training, guidelines, and cohort operation protocols to WCHCs, secondary/tertiary obstetric hospitals, and community healthcare centers. The study was approved by the Ethic Committee of Tianjin Women and Children’s Health Center (No. 201706012-1), and was performed in accordance with the ethical standards as laid down in the Declaration of Helsinki and its later amendments or comparable ethical standards. All individuals were recruited with their voluntary participation. Before enrollment, each participant was given detailed explanation about the study by a professional clinician, and written informed consent was obtained from each participant.

Participant enrollment commenced in August 2017 and planned to complete the recruitment of 10,000 families within 4 years. The recruitment criteria included: (1) pregnant women older than 18 years; (2) at ≤14^+6^ gestational week (pregnant women who registered at primary healthcare centers at ≤12^+6^ gestational week were informed about the cohort, and women who were willing to participate the study were required to come to the WCHCs for formal recruitment and baseline investigation within 2 weeks); (3) women and their spouses need to be registered as Chinese citizens, and have permanent residences in Tianjin; (4) intend to complete antenatal care and give birth in Tianjin, and intend to stay in Tianjin at least until the children are 3 years old; and (5) agree to participate the TJBC study and fulfill the requirement of follow-up medical assessment and biological sample collection.

**Figure 2.**
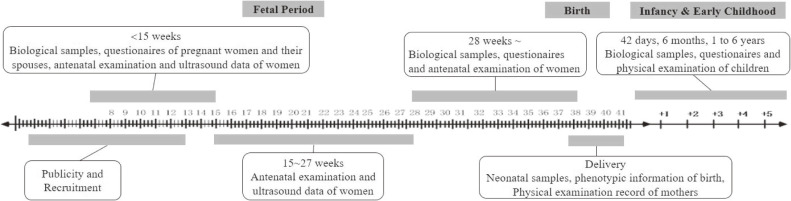
Follow up strategies and workflow of data collection

Follow-up strategies are illustrated in Figure 2. Baseline investigation was conducted at <15 gestational weeks, with biological samples and questionnaires collected from the pregnant women and their spouses, and antenatal examination records collected from each woman. Follow-up investigation was conducted at the mid- and late-pregnancy, delivery, 42 days after birth, 6 months after birth, and each year until 6 years old. Women’s biological samples, questionnaires and antenatal examination records were collected at 15–27 gestational weeks and at 28–41 gestational weeks, respectively, with intervals greater than 4 weeks. At delivery, neonatal samples and healthcare records were collected. At each stage after birth, children samples and physical examination results were collected, and questionnaires were obtained from their parents.

**Figure 3.**
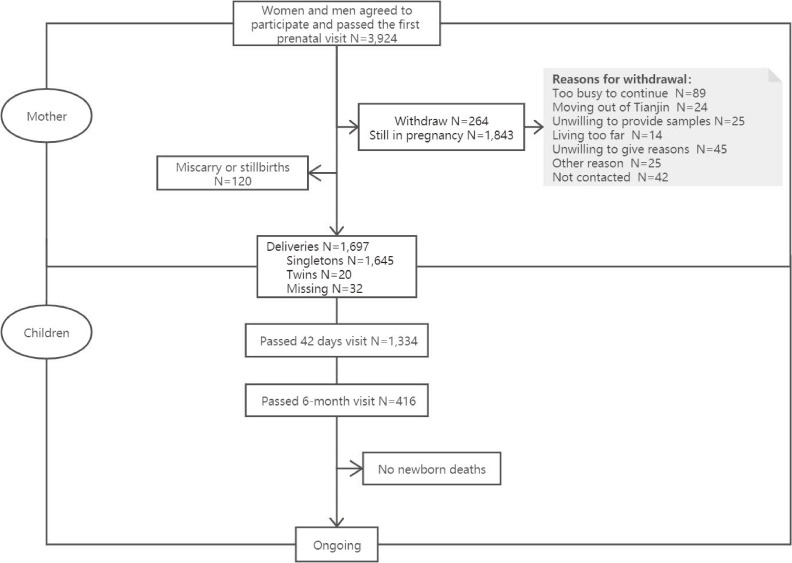
Flow chart of cohort participant recruitment and follow-up

The flow chart of cohort participant recruitment and follow-up is illustrated in Figure 3 (as of January 27, 2019). Among 3,924 pregnant women enrolled in the TJBC, a total of 264 women (6.7%) were lost to follow-up. From August 2017 to December 2017, 9 women were lost to follow-up, 211 women were lost to follow-up in 2018, and 44 women were lost to follow-up in January 2019; the cumulative rate of lost to follow-up was 2.2%, 6.1%, and 6.7% in 2017, 2018, and 2019, respectively. As shown in Table 1, comparing with those who remained in the study, women who opted out were more likely to have smaller pre-pregnancy body mass index (BMI), higher level of education, and higher proportion of being technicians and living in urban areas.

**Table 1.** Baseline characteristics of women who have withdrawn consent and who continue in the study

Baseline characteristics	Remained in the cohort	Consent withdrawn	*P*-value
*N*	3,660	264	
Age, years, mean (SD)	30.31 (3.94)	30.41 (3.88)	0.868^a^
Early-pregnancy BMI, kg/m^2^, mean (SD)	23.13 (3.93)	22.29 (3.64)	0.001^a^
Occupation, *n* *(%)*			
Administrator or manager	207 (5.7)	23 (8.7)	0.023^b^
Technician	1,078 (29.8)	96 (36.8)	
Office worker	1,038 (28.7)	61 (23.4)	
Commerce or service industry	497 (13.7)	31 (11.9)	
Farmer or manufactory worker	216 (6.0)	8 (3.1)	
Unemployed	556 (15.4)	41 (15.5)	
Others	23 (0.6)	1 (0.4)	
Education level, *n* *(%)*			
Middle school or lower	161 (4.5)	7 (2.7)	0.018^b^
High school	359 (9.9)	16 (6.1)	
Vocational/technical college	974 (26.9)	58 (22.2)	
Undergraduate degree	1,737 (48.0)	145 (55.6)	
Graduate school or higher	385 (10.6)	35 (13.4)	
Permanent address, *n* *(%)*			
Urban areas	2,599 (72.9)	227 (90.4)	0.000^b^
Suburban areas	960 (26.9)	23 (9.2)	
Rural area	6 (0.2)	1 (0.4)	
Pre-pregnancy smoking, *n* *(%)*			
Yes	149 (4.1)	13 (5.0)	0.502^b^
No	3,467 (95.9)	248 (95.0)	
Current smoking, *n* *(%)*			
Yes	34 (0.9)	3 (1.1)	0.736^b^
No	3,582 (99.1)	258 (98.9)	
Pre-pregnancy passive smoking, *n* *(%)*			
Yes	1,314 (36.3)	86 (33.0)	0.272^b^
No	2,303 (63.7)	175 (67.0)	
Current passive smoking, *n* *(%)*			
Yes	336 (9.3)	30 (11.5)	0.239^b^
No	3,281 (90.7)	231 (88.5)	
Current alcohol drinking, *n* *(%)*			
Yes	87 (2.4)	2 (0.8)	0.088^b^
No	3,530 (97.6)	259 (99.2)	
Pre-pregnancy tea drinking, *n* *(%)*			
Yes	1,926 (53.4)	123 (47.5)	0.068^b^
No	1,684 (46.6)	136 (52.5)	
Current tea drinking, *n* *(%)*			
Yes	201 (5.6)	11 (4.3)	0.372^b^
No	3,406 (94.4)	247 (95.7)	
Pre-pregnancy coffee drinking, *n* *(%)*			
Yes	1,703 (47.1)	121 (46.9)	0.945^b^
No	1,911 (52.9)	137 (53.1)	
Current coffee drinking, *n* *(%)*			
Yes	79 (2.2)	6 (2.3)	0.886^b^
No	3,528 (97.8)	252 (97.7)	

BMI, body mass index; SD, standard deviation.

^a^Mann-Whitney U-test, ^b^chi-square test.

### Sample size and power calculations

The overall TJBC sample size target is 10,000 families, which provides adequate power to detect moderately strong causal effects of common environmental, social and biological exposures. According to previous reports in the Chinese population, we assumed the incidence rates of GDM was 9.3%,^13^ preterm birth was 7.2%,^26^ birth defects was 5.6%,^27^ childhood obesity was 16.3%,^14^ and childhood hypertension was 9.0%.^28^ With those incidence rates, we estimated the smallest detectable odds ratios based on 80% power at a testing level of 0.05 in the TJBC cohort (as shown in eTable 1).

### Data collection and measurement

Semi-structured questionnaires were designed to collect socio-demographics, environmental exposures, lifestyle behaviors, diet, psychological evaluation, and childhood development, once or repetitively (Table 2). All questionnaires were completed with face-to-face guidance by designated clinicians to ensure the integrity and credibility of answers. Marital Satisfaction Scale, part of the ENRICH marital inventory, was used to judge the happiness of marriage.^15^ The individual’s perceived social support within the family and outside the family was evaluated using the Chinese versions of the Perceived Social Support Scale (PSSS).^16^ Mental health (depression and anxiety) during pregnancy was assessed using a 20-item self-rating depression scale (SDS) and a 20-item self-rating anxiety scale (SAS).^17^^,^^18^

**Table 2.** Questionnaires data collection of women, men, and their children in TJBC

Questionnaires	Participants	phases
**Demographic characteristics**		
Occupation/employment	Women and Men	Early pregnancy
Education		
Gestational age at birth, Number of fetus, ​ Birth weight		
Permanent address		
Family members		

**Gestation related information**		
Age of first menstruation	Women	Early pregnancy
Planned pregnancy		
Pregnancy way		
Reproductive history		

**Mental health**		
Marital Satisfaction Scale	Women	Mid-pregnancy
Perceived Social Support Scale		Mid-pregnancy
Self-rating Depression Scale		Mid-pregnancy, Late pregnancy
Self-rating Anxiety Scale		Mid-pregnancy, Late pregnancy
Pressure, negative emotion		Mid-pregnancy, Late pregnancy, 42 days after birth, at the age of 6 months, 1 years

**Dwelling environment**		
Pets	Family	Early pregnancy, at the age of 6 months, 1 to 6 years
	
Decoration		Early pregnancy
Mould		
Lampblack		
Frozen		

**Health**		
Toxic and hazardous exposure	Women	Early pregnancy, Late pregnancy
	Men	Early pregnancy
Family medical history	Women, Men	Early pregnancy
Vaginal bleeding during pregnancy	Women	Late pregnancy
Medical history	Women, Men	Early pregnancy
	Children	42 days after birth, at the age of 6 months, 1 to 6 years
Health status	Women	Mid-pregnancy, Late pregnancy, 42 days after birth, at the age of 6 months, 1 year
	Children	42 days after birth, at the age of 6 months, 1 to 6 years
Medications	Women	Mid-pregnancy, Late pregnancy
	Children	42 days after birth, at the age of 6 months, 1 to 6 years
Antibiotics	Children	42 days after birth, at the age of 6 months, 1 to 6 years
Allergies		
Family history of allergic diseases	Children	42 days after birth
Type of allergic diseases		at the age of 6 months, 1 to 6 years

**Lifestyle**		
Passive smoking status	Children	42 days after birth, at the age of 6 months, 1 to 6 years
Smoking status, passive smoking status	Women	Early pregnancy, Late pregnancy
	Men	Early pregnancy
Alcohol	Women	Early pregnancy, Late pregnancy
	Men	Early pregnancy
Tea, coffee, carbonated drinks	Women	Early pregnancy, Late pregnancy
	
Drinking water		Early pregnancy
Sleep	Women	Early pregnancy, Mid-pregnancy, Late pregnancy, 42 days after delivery, at the age of 6 months, 1 years
	Children	42 days after birth, at the age of 6 months, 1 to 6 years
Physical activity	Women	Early pregnancy, Mid-pregnancy, Late pregnancy,
	Children	42 days after birth, at the age of 6 months, 1 to 6 years

**Micronutrient supplements**	Women	Early pregnancy, Late pregnancy
	Children	42 days after birth, at the age of 6 months, 1 to 6 years

**Diet**		
Dietary changes	Women	Mid-pregnancy
Place		
Food frequency questionnaire		
	
Breastfeeding	Children	42 days after birth, at the age of 6 months, 1 year
Milk or formula feeding		42 days after birth, at the age of 6 months, 1 to 3 years
Food frequency/preference		at the age of 6 months, 1 to 6 years
Eating behavior		at the age of 6 months, 1 to 6 years

**Parent-child communication**	Children	42 days after birth

**Child development**	Children	at the age of 6 months, 1 to 6 years

TJBC, Tianjin Birth Cohort.

Clinical information of antenatal and postnatal healthcare was collected from the hospital healthcare system, including antenatal screening test results, ultrasound findings, obstetric complications, clinical treatments and medications, and anthropometry results at each follow-up phase (Table 3).

**Table 3.** Clinical data collection of women, men, and their children in TJBC

Clinical data	Participants	phases
Height	Women, Men	Early pregnancy
Weight	Men	Early pregnancy
	Women	Early pregnancy, Before delivery, 42 days after delivery
Clinical test results	Women	Early pregnancy, Mid-pregnancy, Late pregnancy
Ultrasound measurements		Mid-pregnancy, Late pregnancy
Pregnancy complications (eg, GDM, PIH)		Mid-pregnancy
Delivery records (eg, date, mode)		At delivery

Gender	Children	At birth
Date of birth		At birth
Birth weight/length, Apgar score		At birth
Height		At birth, 42 days after birth, at the age of 6 months, 1 to 6 years
Weight		At birth, 42 days after birth, at the age of 6 months, 1 to 6 years
Clinical test results		at the age of 3 to 6 years
Medication		at the age of 3 to 6 years
Diagnosis		at the age of 3 to 6 years
Intellectual development assessment		at the age of 1 to 2 years

GDM, gestational diabetes mellitus; PIH, pregnancy-induced hypertension; TJBC, Tianjin Birth Cohort.

Newborn birth data was obtained from the delivery records. In early childhood, we collected clinical assessment, physical measurements (eg, weight, body length/height), clinical examination using routine state health checks at 6 weeks and 6, 12, and 36 months of age, all of which were conducted by experienced medical staff. Further clinical information including clinical test results, intellectual development assessment, medical history, and disease diagnosis were collected through record linkage to children’s medical information.

A variety of biological samples, including blood, urine, feces, and breast milk, were collected at each follow-up phase from the families participating in this cohort by clinicians (Table 4). At delivery, placental tissues, umbilical cord blood, and umbilical cord were collected from the two selected hospitals with good conditions of sample collection and quality control. Pregnant women were closely followed in case of adverse pregnancy outcomes such as miscarriage, stillbirth, and birth defects. For such cases, products of conception (POC) were collected when possible. All samples were collected with individual tubes, each sample tube was supplied with a unique barcode associating with the sample owner’s personal identity. After collection, whole blood was separated as plasma and buffy coat within 8 hours. Umbilical cord blood was aliquoted in 24 hours. Breast milk samples were aliquoted within 8 hours. All samples were temporarily stored at −80°C in Tianjin, and periodically transported to the CNGB, which is certified to ISO 9001, ISO 14001, ISO/OHSAS 18001, and ISO 27001, for long-term storage at −80°C. Beijing Genomics Institute at Shenzhen (BGI-Shenzhen) was responsible for providing technical assistance, IT support, and genomics research facilities for the TJBC study. Samples collection progress is shown in eTable 2.

**Table 4.** Sample collection of women, men, and their children in TJBC

Role	Sample type	During pregnancy			delivery	After delivery						
	
		<15 weeks	15–27 weeks	28–41 weeks		42 days	6 months	1 year	2 years	2–3 years	4–5 years	5–6 years
Mother	Whole Blood	✓	✓	✓								
	Urine	✓	✓	✓								
	Faeces	✓	✓	✓								
	Abortion Tissue	If have, ✓										
	Placenta				✓							
	Umbilical Cord Blood				✓							
	Umbilical Cord				✓							
	Breast milk					✓	✓					
Father	Whole blood	✓										
child	Dried blood spots					✓						
	Fingertip blood									✓	✓	✓
	Urine							✓	✓	✓	✓	✓
	Faeces							✓	✓	✓	✓	✓

TJBC, Tianjin Birth Cohort.

### Data analysis

Descriptive statistics were used to summarize baseline characteristics. Mann-Whitney U-test was conducted as univariate analysis to identify continuous variables influencing follow-up. Categorical variables were presented as numeric and were compared using chi-square test. Confidence intervals (CIs) were presented as 95% CIs and *P*-values less than 0.05 were deemed statistically significant. Statistical analysis was performed using SPSS 24.0 (IBM, Chicago, IL, USA).

## RESULTS

As of January 2019, a total of 3,924 pregnant women were enrolled. As compared with the target population of 34,924 pregnant women (summarized by the number of women who set up pregnancy records in each district, from the specific date when enrollment started, to January 2019), the participation rate was 11.2%. Baseline characteristics are shown in Table 1. Maternal age was 30.3 (standard deviation [SD], 3.9) years, and early-pregnancy BMI was 23.1 (SD, 3.9) kg/m^2^. Around 30% of women work as technicians, and 28.7% as office workers. In terms of education level, 58.6% of women had undergraduate or higher degrees, and 26.9% had vocational or high school education. A total of 72.9% participants was living in urban areas. Very few women smoked (4.1% of pre-pregnancy vs 0.9% of current) or drank (2.4%) during pregnancy. Nearly half of women passively smoked (36.3%), drank tea (53.4%), and drank coffee (47.1%) before pregnancy, but significantly decreased during pregnancy.

Among 3,924 pregnant women, there were 119 (3.0%) miscarriages. Among 1,665 women who had live birth (Table 5), the prevalence of gestational diabetes mellitus (GDM) was 18.1%, anemia was 20.4%, thyroid hypofunction was 2.0%, uterine fibroids was 5.9%, hypertension was 1.9%, ovarian cyst was 2.9%, scarred uterus was 1.7%, vulvovaginal candidiasis was 1.1%, urinary system infection was 0.9%, hepatitis B virus was 0.8%, and maternal-child blood incompatibility as 0.2%. Among the 1,645 singleton births, 92 (5.6%) were preterm birth (PTB), 61 (3.7%) were low birth weight, and 120 (7.3%) were macrosomia.

**Table 5.** Diseases and adverse birth outcomes of participants

Diseases and adverse birth outcomes	*N* *(%)*	Total
Miscarriage	119 (3.0)	3,924

Gestational diabetes mellitus	302 (18.1)	1,665
Anemia	340 (20.4)	
Thyroid hypofunction	33 (2.0)	
Uterine fibroids	98 (5.9)	
Hypertension	31 (1.9)	
In-vitro fertilization	30 (1.8)	
Ovarian cyst	48 (2.9)	
Scarred uterus	28 (1.7)	
Vulvovaginal candidiasis	18 (1.1)	
Urinary system infection	15 (0.9)	
Hepatitis B virus	13 (0.8)	
Maternal-child blood incompatibility	4 (0.2)	

Preterm birth	92 (5.6)	1,645
Low birth weight	61 (3.7)	
Macrosomia	120 (7.3)	

Birth defects	9 (0.5)	1,685
Congenital heart disease	3	
Other deformities of the outer ear (small ears/no ears)	2	
Rectal anal atresia or stenosis (including no anus)	1	
Cleft palate	1	
Syndactyly	1	
Multi-finger	1	

The PTB rate in the current study was lower than the global average rate (10.6%)^19^ but similar with other studies in China.^9^^,^^10^ By constructing logistic regression models, we identified that paternal age (OR 1.07; 95% CI, 1.01–1.14 for each year increase), vaginal bleeding during pregnancy (OR 2.82; 95% CI, 1.54–5.17) and maternal early-pregnancy BMI (OR 1.08; 95% CI, 1.01–1.15 for each kg/m^2^ increase) were factors that increased the risk of PTB.

## DISCUSSION

The main strengths of the TJBC include: (1) The TJBC study has the strength of collecting comprehensive maternal, paternal, and childhood information (not only maternal but also paternal data is collected, which overcomes the shortcomings of traditional maternal-child birth cohorts), with the potential for causal link studies between early-life exposures and later health conditions. (2) Additionally, the TJBC study has the strength of a diverse range of biological samples, and we are actively engaging with technical advances and rapid development of emerging new technologies, including metabolomics, epigenomics, metagenomics, and immunomics.

Several limitations of this study should be noted. (1) The cohort covers urban and suburban populations of Tianjin local residents, but with almost no rural participants included. Thus the findings of the cohort may have difficulty in extrapolating to rural populations. (2) The sample size is large for causal link researches of early-life exposures with health consequences, but not as large as several mega birth cohorts. The TJBC will continue to recruit participants and the follow-up will be extended to adolescence.

In conclusion, the current paper provides a profile of a large-scale prospective birth cohort in northern China. The TJBC collects comprehensive maternal, paternal, and childhood information, including a diverse range of biological samples, with the potential for multi-omics research, which would provide new insight on the causal link between early-life exposures and later health conditions.

## APPENDIX A. SUPPLEMENTARY DATA

eTable 1. Smallest detectable odds ratios (ORs) in a case-control analysis

eTable 2. Samples collection progress

## References

[1] He C, Liu L, Chu Y, . National and subnational all-cause and cause-specific child mortality in China, 1996–2015: a systematic analysis with implications for the Sustainable Development Goals. Lancet Glob Health. 2017;5:e186–e197. 10.1016/S2214-109X(16)30334-5 28007477 PMC5250590

[2] Gao Y, Zhou H, Singh NS, . Progress and challenges in maternal health in western China: a Countdown to 2015 national case study. Lancet Glob Health. 2017;5:e523–e536. 10.1016/S2214-109X(17)30100-6 28341117 PMC5387688

[3] Batis C, Sotres-Alvarez D, Gordon-Larsen P, Mendez MA, Adair L, Popkin B. Longitudinal analysis of dietary patterns in Chinese adults from 1991 to 2009. Br J Nutr. 2014;111:1441–1451. 10.1017/S0007114513003917 24331247 PMC3966951

[4] Zhou M, Wang H, Zhu J, . Cause-specific mortality for 240 causes in China during 1990–2013: a systematic subnational analysis for the Global Burden of Disease Study 2013. Lancet. 2016;387:251–272. 10.1016/S0140-6736(15)00551-6 26510778

[5] Zhou Z, Zheng D, Wu H, . Epidemiology of infertility in China: a population-based study. BJOG. 2018;125:432–441. 10.1111/1471-0528.14966 29030908

[6] Dong Y, Jan C, Ma Y, . Economic development and the nutritional status of Chinese school-aged children and adolescents from 1995 to 2014: an analysis of five successive national surveys. Lancet Diabetes Endocrinol. 2019;7:288–299. 10.1016/S2213-8587(19)30075-0 30902266

[7] Barker DJ, Osmond C. Infant mortality, childhood nutrition, and ischaemic heart disease in England and Wales. Lancet. 1986;1:1077–1081. 10.1016/S0140-6736(86)91340-1 2871345

[8] Gillman MW, Barker D, Bier D, . Meeting report on the 3rd International Congress on Developmental Origins of Health and Disease (DOHaD). Pediatr Res. 2007;61:625–629. 10.1203/pdr.0b013e3180459fcd 17413866

[9] Qiu X, Lu JH, He JR, . The Born in Guangzhou Cohort Study (BIGCS). Eur J Epidemiol. 2017;32:337–346. 10.1007/s10654-017-0239-x 28321694

[10] Zhang J, Tian Y, Wang W, . Cohort profile: the Shanghai Birth Cohort. Int J Epidemiol. 2019;48:21–21g. 10.1093/ije/dyy277 30629180

[11] Tao FB, Hao JH, Huang K, . Cohort Profile: the China-Anhui Birth Cohort Study. Int J Epidemiol. 2013;42:709–721. 10.1093/ije/dys085 22729236

[12] National Bureau of Statistics of China. Statistical Yearbook of China (in Chinese). http://www.stats.gov.cn/tjsj/ndsj/. Accessed Sep 12, 2019.

[13] Leng J, Shao P, Zhang C, . Prevalence of gestational diabetes mellitus and its risk factors in Chinese pregnant women: a prospective population-based study in Tianjin, China. PLoS One. 2015;10:e0121029. 10.1371/journal.pone.0121029 25799433 PMC4370728

[14] Wang S, Dong YH, Wang ZH, Zou ZY, Ma J. Trends in overweight and obesity among Chinese children of 7–18 years old during 1985–2014. Chin J Prev Med. 2017;51:300–305. 10.3760/cma.j.issn.0253-9624.2017.04.005 28395462

[15] Fowers BJ, Olson DH. Enrich marital inventory: a discriminant validity and cross-validation assessment. J Marital Fam Ther. 1989;15:65–79. 10.1111/j.1752-0606.1989.tb00777.x 21118433

[16] Jiang QJ. Perceived social support scale. Chin J Behavioral Med Sci. 2001:41–42.

[17] Shu L. Self-Rating Depression Scale. Chin Ment Health J. 1993:194–196.

[18] Wu WY. Self-Rating Anxiety Scale. Chin Ment Health J. 1993:235–238.

[19] Chawanpaiboon S, Vogel JP, Moller AB, . Global, regional, and national estimates of levels of preterm birth in 2014: a systematic review and modelling analysis. Lancet Glob Health. 2019;7:e37–e46. 10.1016/S2214-109X(18)30451-0 30389451 PMC6293055

[20] Yang S, Mei H, Mei H, . Risks of maternal prepregnancy overweight/obesity, excessive gestational weight gain, and bottle-feeding in infancy rapid weight gain: evidence from a cohort study in China. Sci China Life Sci. 2019;62:1580–1589. 10.1007/s11427-018-9831-5 31745693

[21] Yang X, Tian H, Zhang F, . A randomised translational trial of lifestyle intervention using a 3-tier shared care approach on pregnancy outcomes in Chinese women with gestational diabetes mellitus but without diabetes. J Transl Med. 2014;12:290. 10.1186/s12967-014-0290-2 25349017 PMC4213554

[22] Schüssler-Fiorenza Rose SM, Contrepois K, Moneghetti KJ, . A longitudinal big data approach for precision health. Nat Med. 2019;25:792–804. 10.1038/s41591-019-0414-6 31068711 PMC6713274

[23] Perkins BA, Caskey CT, Brar P, . Precision medicine screening using whole-genome sequencing and advanced imaging to identify disease risk in adults. Proc Natl Acad Sci USA. 2018;115:3686–3691. 10.1073/pnas.1706096114 29555771 PMC5889622

[24] Zhu Y, Li M, Rahman ML, . Plasma phospholipid n-3 and n-6 polyunsaturated fatty acids in relation to cardiometabolic markers and gestational diabetes: A longitudinal study within the prospective NICHD Fetal Growth Studies. PLoS Med. 2019;16:e1002910. 10.1371/journal.pmed.1002910 31518348 PMC6743768

[25] Koren O, Goodrich JK, Cullender TC, . Host remodeling of the gut microbiome and metabolic changes during Pregnancy. Cell. 2012;150:470–480. 10.1016/j.cell.2012.07.008 22863002 PMC3505857

[26] Guo T, Wang Y, Zhang H, . The association between ambient temperature and the risk of preterm birth in China. Sci Total Environ. 2018;613–614:439–446. 10.1016/j.scitotenv.2017.09.104 28918275

[27] Yu M, Ping Z, Zhang S, He Y, Dong R, Guo X. The survey of birth defects rate based on birth registration system. Chin Med J (Engl). 2015;128:7–14. 10.4103/0366-6999.147785 25563306 PMC4837823

[28] Zhao Y, Wang L, Xue H, Wang H, Wang Y. Fast food consumption and its associations with obesity and hypertension among children: results from the baseline data of the Childhood Obesity Study in China Mega-cities. BMC Public Health. 2017;17:933. 10.1186/s12889-017-4952-x 29212483 PMC5719642

