# Supplementary material for: Study Design and Baseline Profiles of Participants in the Tianjin Birth Cohort (TJBC) in China
Source: J Epidemiol. 2022 Jan 5;32(1):44–52. doi: 10.2188/jea.JE20200238 (PMC8666315; doi:10.2188/jea.JE20200238)
Supplement: Supplementary file 1 [file je-32-044-s001.pdf]

eTable 1. Smallest detectable odds ratios (ORs) in a case-control analysis

Outcome	Incidence rates (%)	Follow-up rates (%)	Exposure prevalence among controls		
			5%	10%	20%
GDM	9.3	95	1.56	1.38	1.28
Preterm birth	7.2	95	1.65	1.44	1.32
Birth defects	5.6	90	1.78	1.53	1.38
Childhood obesity	16.3	80	1.46	1.32	1.23
Childhood hypertension	9	80	1.63	1.43	1.31

GDM, gestational diabetes mellitus.

eTable 2. Samples collection progress

Role	Sample type	N (%)
Mother	Whole Blood	8,742 (97.9)
	Urine	8,680 (97.2)
	Feces	8,068 (91.6)
	Abortion Tissue	14 (11.8)
	Placenta	128 (7.7)
	Umbilical Cord Blood	125 (7.5)
	Umbilical Cord	128 (7.7)
Father	Whole blood	3,773 (96.4)
Child	Dried blood spots	540 (67.1)
	Urine	32 (1.9)
	Feces	676 (69.6)
